# A Smart Tendon Hammer System for Remote Neurological Examination

**DOI:** 10.3389/frobt.2021.618656

**Published:** 2021-03-16

**Authors:** Waiman Meinhold, Yoshinori Yamakawa, Hiroshi Honda, Takayuki Mori, Shin-ichi Izumi, Jun Ueda

**Affiliations:** ^1^ Biorobotics and Human Modeling Laboratory, Woodruff School of Mechanical Engineering, Georgia Institute of Technology, Atlanta, GA, United States; ^2^ NITI-ON Co. Ltd., Funabashi, Japan; ^3^ Graduate School of Biomedical Engineering, Tohoku University, Sendai, Japan

**Keywords:** remote diagnosis, neurology, IoT, reflex, COVID-19 pandemic

## Abstract

The deep tendon reflex exam is an important part of neurological assessment of patients consisting of two components, reflex elicitation and reflex grading. While this exam has traditionally been performed in person, with trained clinicians both eliciting and grading the reflex, this work seeks to enable the exam by novices. The COVID-19 pandemic has motivated greater utilization of telemedicine and other remote healthcare delivery tools. A smart tendon hammer capable of streaming acceleration measurements wirelessly allows differentiation of correct and incorrect tapping locations with 91.5% accuracy to provide feedback to users about the appropriateness of stimulation, enabling reflex elicitation by laypeople, while survey results demonstrate that novices are reasonably able to grade reflex responses. Novice reflex grading demonstrates adequate performance with a mean error of 0.2 points on a five point scale. This work shows that by assisting in the reflex elicitation component of the reflex exam via a smart hammer and feedback application, novices should be able to complete the reflex exam remotely, filling a critical gap in neurological care during the COVID-19 pandemic.

## 1 Introduction

The deep tendon reflex (DTR) is a critically important diagnostic tool for multiple upper and lower neuron neurological illnesses Chardon et al. (2014); Walker et al. (1990). Assessment of the DTR is often the first step toward localization of a neurological lesion. Crucially, the DTR exam requires physical interaction with a patient, and is thus limited when in-person healthcare delivery is reduced.

The COVID-19 pandemic has strained existing healthcare resources while simultaneously providing significant impetus for the development and implementation of remote diagnostic and therapeutic systems for healthcare delivery Chauhan et al. (2020). The development of a system for smart delivery of tendon tapping stimulation will aid in the remote assessment of deep tendon reflexes (DTRs) with future potential for therapeutic applications as well. Although there have been reported neurological symptoms of COVID-19 Ottaviani et al. (2020); Pezzini and Padovani (2020), the primary effect to neurological diagnosis and treatment is likely the backlog of urgent, non-COVID-19 related healthcare needs.

Examination of the DTR is a standard part of the neurological exam, but one that requires both physical contact between the clinician and patient, and the use of a non-disposable medical hammer. The assessment of DTRs requires two main steps, 1) delivery of adequate stimulus via tendon tapping, and 2) grading of the reflex response. Clinicians receive training in both of these skills, but the stimulus delivery is the component that must be performed in close physical proximity to the patient.

The DTR involves afferent neurons in the muscle that synapse directly onto the motor neurons of that muscle. The reflex is stimulated via the application of an impact from a reflex hammer. The hammer impact displaces the tendon, lengthening the muscle tendon complex, and stretching the afferent neurons in the muscle fibers Walker et al. (1990). This stretching of the neurons activates the reflex loop, causing a rapid contraction of the muscle and rotation of the joint. The reflex response is then graded on a numerical scale, either five point (National Institute of Neurological Disorders and Stroke), or nine point (Mayo) Manschot et al. (1998).

A number of conditions such as hypothyroidism, peripheral neuropathy, myoclonus and parkinsonism affect the reflex response Chardon et al. (2014); Walker et al. (1990); Eslamian et al. (2011). Efforts to quantify the dynamics of various DTR’s are ongoing Shaik et al. (2020); Mamizuka et al. (2007); Manschot et al. (1998); Kim et al. (2002); Chardon et al. (2014); Stam and Tan (1987); Frijns et al. (1997), however, typical diagnostic use is as a screening tool to indicate the presence of lesion and aid in localization in either the upper or lower reflex arc, Walker et al. (1990).

While laypeople may be able to accurately grade reflexes, stimulation of the tendon with a reflex hammer is more difficult. To that end, this work involves the development of a smart tendon hammer and accompanying application for the immediate assessment of tendon tapping stimulus. By measuring hammer acceleration during tapping, it is possible to characterize the stimulus as appropriate or inappropriate (in terms of tapping location) after each individual tap. Implementation of this categorization in a mobile application enables tendon stimulus delivery by a layperson, as the skilled component of the procedure then becomes the response grading. The reflex grading portion of the proposed remote tendon exam is validated through a video response survey in which both novice and expert participants grade reflexes from video segments.

## 2 Materials and Methods

**FIGURE 1:**
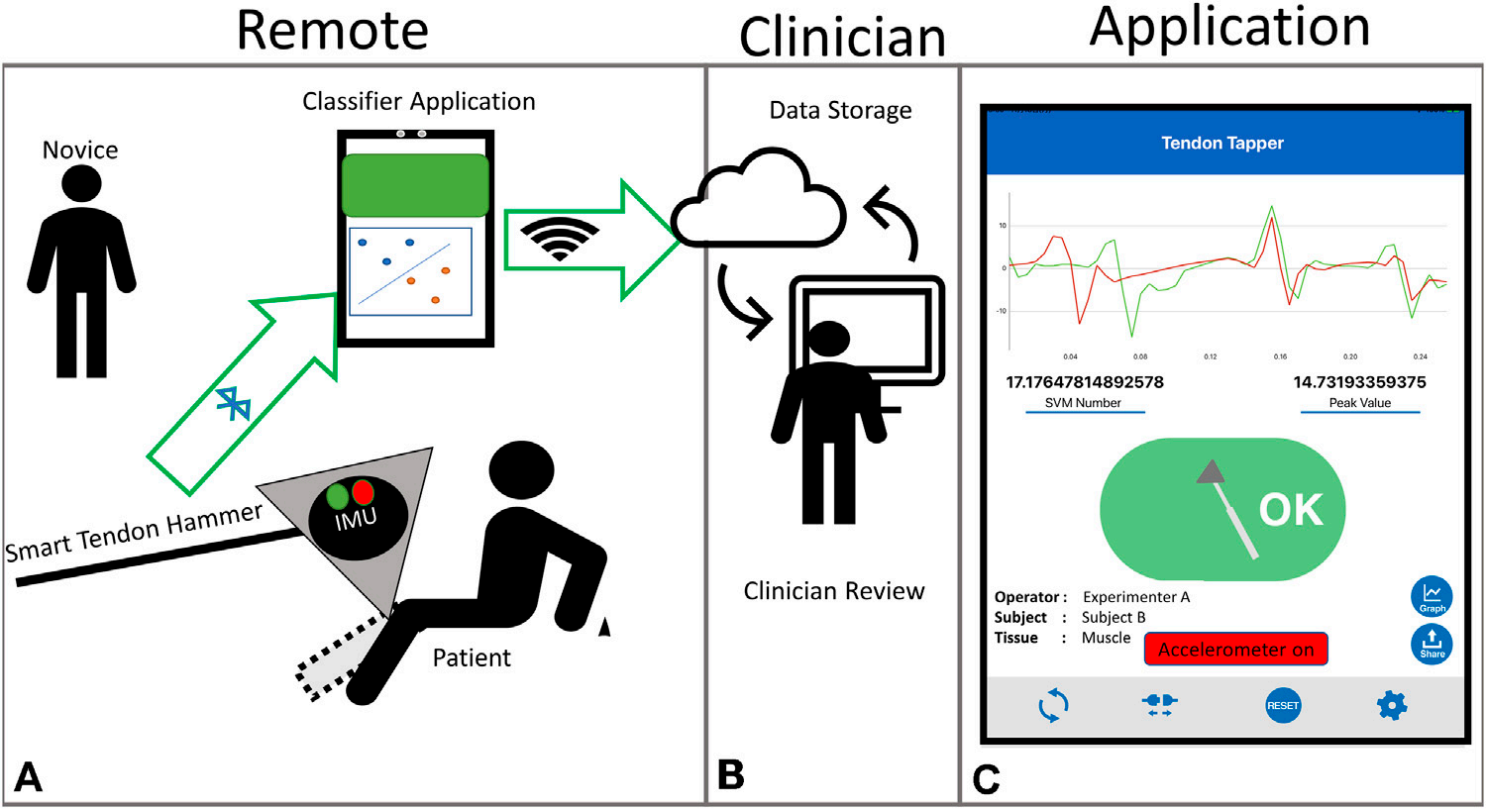
Proposed remote deep tendon reflex exam system **(A)** Remote portion with smart hammer assisted reflex elicitation and grading, **(B)** Clinical portion, with clinician receiving assessed scores and communicating with patient, **(C)** Tapping application screen.

All of the work, experiments and results reported here aim to provide motivation and validation of the remote DTR evaluation workflow and smart hammer system shown in Figure 1. The system is intended to enable physical separation between patient and clinician, with a patient’s caretaker or family member serving as the novice participant delivering the tendon tapping and grading the reflex response. A smart hammer streams tapping accelerations to the mobile application, which then provides feedback to the operator about the tapping location. Once grades are recorded, they can be sent along with the associated tapping data to a clinician for review.

This method of remote tapping contains key differences from the standard tapping procedure that clinicians perform. In the traditional clinical tapping by an expert, this characterization of stimulus is done through experience and “feel”, primarily based on visual location of the impact site and the rebounding of the hammer in the clinician’s hand. The proposed system utilizes the smart hammer to collect and analyze the hammer acceleration in order to provide the same characterization of stimulus to a novice who lacks the experience and training of an expert.

First, tapping classification via acceleration is assessed for feasibility and performed via support vector machine (SVM). Next, the design and development of an assistive application for tendon tapping feedback is presented. Novice tapping variability is compared to expert performance in terms of impact acceleration viability. Finally, novice reflex grading is compared to expert grading through the use of an online video reflex grading task.

### 2.1 Tapping Classification

The primary aim of the technical developments reported here is the accurate classification of tendon tapping from hammer acceleration measurements. It is essential that the information to discriminate between correct and incorrect stimulation is contained within the readily measurable hammer acceleration. In the case of the wireless instrumented hammer and mobile application, Bluetooth communication is the primary limiting factor for the sample rate. To evaluate the practicality of classification via streamed acceleration measurements (limited to 200 Hz), human tapping data was collected at 800 Hz using a previously developed automated tapping device Meinhold and Ueda (2018). 50 taps were analyzed from two locations on each of the two subjects, the apex of the right Achilles tendon, and an adjacent location. All data collection occurred under an institutionally approved protocol (GT# H17264).

**FIGURE 2:**
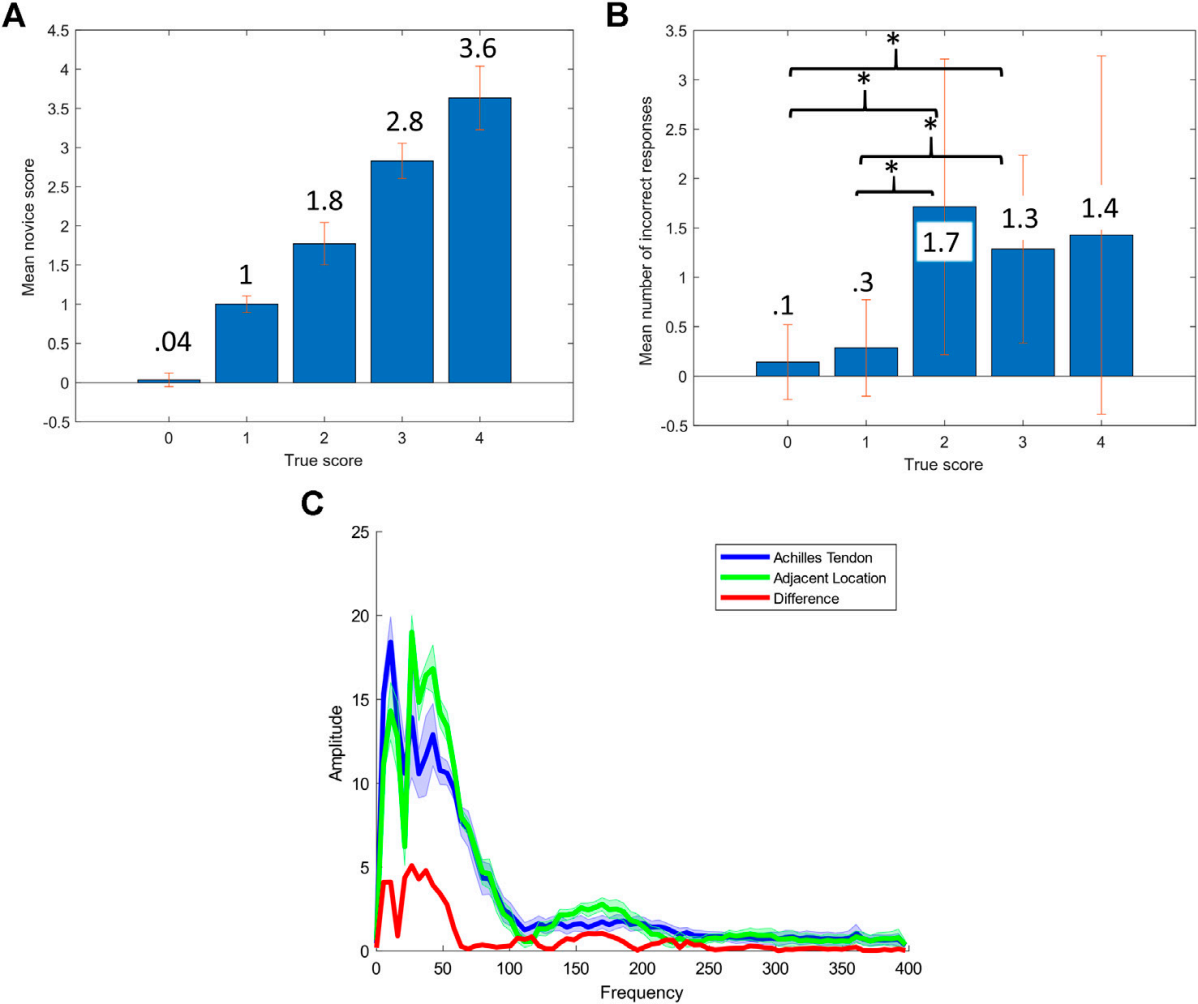
Survey and automated tapping results **(A)** Mean score for each true score, **(B)** Mean number of errors for each true score, * represents p<0.05. **(C)** Mean amplitude spectrum of robotic tapping on two human subjects, shaded portions represent one standard deviation.

The frequency content of each tap was ascertained by discrete Fourier transform (*fft*, MATLAB, MathWorks Inc., United States). Mean results for both subjects, along with the difference between locations, are shown in Figure 2C. Clear separation between the tendon and incorrect location is apparent up to about 50 Hz, which indicates that the 200 Hz sampling rate may be sufficient for tapping classification. A *t*-test confirmed (p<0.001) that the mean power of the first 50 Hz of the spectrum was significantly different between the on tendon and off tendon conditions. There was a significant difference (p<0.01) in the mean power from 50 to 400 Hz, but not from 100 to 400 Hz (p>0.05). For the statistical tests, power in the respective bandwidths was computed for each tap, then the groups of 100 were tested against the null hypothesis that there was not a difference in the population means. The results of a $\chi^{2}$ test for the full frequency range are shown in the Supplementary Material. Although the reported results pertain only to the Achilles tendon, it is expected that other tendons produce similar acceleration profiles and frequency spectra.

Both the waveform and amplitude of the frequency response contain important information for differentiating the two locations. The waveform most likely contains differences in frequency dependent damping, while differences in amplitude are most likely due to the level of impedance matching between the hammer and tissues. These properties are each individually viable choices for location classification, while acceleration time series contains both frequency and amplitude information, such that any classifier utilizing acceleration time series inherently leverages both domains.

### 2.2 Tendon Hammer Design and Evaluation

A previously developed smart tendon hammer Meinhold et al. (2017) was used in the course of this work. The hammer is a modified commercially available reflex hammer (NITI-ON, Chiba, Japan) with a silicone head, stainless steel handle and wireless Inertial Measurement Unit (IMU) (Mbient Labs, San Francisco, CA, United States) situated in a polymer case in the head. The IMU records 3-axis acceleration at up to 800 Hz for data logged to the onboard memory, or up to 200 Hz for data streamed directly to a mobile device. The addition of the IMU to the commercial hammer requires only the removal of a disc of material out of the silicone head and inserting the IMU case, which can be done without any precision machining. More details on the construction and operation of the instrumented hammer can be found in the previous study Meinhold et al. (2017), as well as the Supplementary Material documentation.

### 2.3 Tapping Variability Comparison

As described above, the force applied by the tendon hammer to the tendon is the mechanism for eliciting the DTR. Stimulation variability is a confounding factor for the diagnostic utility of the procedure. In order to ascertain the potential for laypeople to perform this procedure remotely, manual stimulation variability was compared between an expert and a non-trained operator.

A trained clinician performed a series of 50 taps with the instrumented medical hammer to a latex rubber tendon analog. The tendon analog was used to eliminate variability due to human subject movement or physiological factors. Acceleration was recorded from the embedded sensor at 100 Hz. The repeatability of stimulation intensity was measured and compared. The relative standard deviation (RSD) of peak deceleration during impact was the metric used for comparison.

The performance of a novice operator in the same simulated tapping experiment was also assessed. A series of 50 taps to the surface of the latex rubber tendon was conducted, with the RSD of peak tapping acceleration again being the primary metric of comparison.

### 2.4 Materials Cost and Distribution

The Achilles tapping results and the associated statistical analysis in Section 2.1 demonstrate that sufficient information for classification is contained in the 100 Hz acceleration signal. This has significant benefits for a distributed and remote method of DTR assessment. The 200 Hz bandwidth allows for the use of relatively cheap (61 USD) IMUs as well as standard mobile phone data collection. The process of retrofitting a standard silicone Taylor hammer with an IMU requires only the coarse removal of silicone material to accommodate the IMU case, without any high tolerance machining operations. The ideal tool for communicating with the wireless IMU is a mobile application, as this is easily distributed, and does not require hardware beyond common mobile devices. With distribution of the devices to individuals requiring a DTR exam, physical proximity between patient and clinician is precluded, and sterilization of the hammer between uses is not necessary, because the low cost enables a one device per patient paradigm.

### 2.5 Application Design and Functionality

A preliminary version of this application was previously reported Meinhold et al. (2017). In the past, this application was designed primarily for research users, the version presented here has been adapted to aid novice users. The interface has been streamlined and simplified to avoid confusion. In addition to the tablet interface, LED indicators on the hammer body itself now indicate the success or failure of the prior tap. A diagram of the intended use is shown in Figure 1. The main function of the application is to stream acceleration data from the tendon hammer, detect and classify tendon strikes, then provide binary feedback in the form of a red or green indicator. Although much more information can be recorded, classification results are of the most use to a novice attempting DTR evaluation. The application allows user input of physiological information, as well as import of trained classifiers. These SVM classifiers take the form of a string of parameters defining a hyperplane, that are then used to classify each tap as either on target or incorrect.

### 2.6 SVM Classifier

To evaluate the classification of tendon tapping location from streamed acceleration data, an additional set of human trials were carried out. Each subject underwent 100 taps from the tendon hammer described in Section 2.2, 50 taps to the right Achilles tendon, and 50 taps to a laterally adjacent location. A total of eight healthy adult subjects participated in the experiments (mean age 36.5, 5 F), following an approved human subjects research protocol (GT# H20531).

The large amount of sample points and classification problem associated with traditional stimulus delivery during tendon tapping lends itself to SVM based classification. Although a large number of additional classification methods exist, SVM was chosen due to the portability of the model and the training speed. In order to evaluate the suitability of SVM classification of the tapping location, eight different test/train datasets were produced, with a single subject comprising the test set and the remaining seven making up the training set. The feature vector used consisted of the acceleration at each sample taken by the IMU. A segment of acceleration consisting of the period after impact was used, 0.25 s in length, for a total of 51 samples, a representative feature vector is shown in the Supplementary Material document. Each acceleration time series (feature vector) was standardized prior to training of the model. The models were trained in MATLAB (fitsvm), with a linear kernel. Accuracy of the models was assessed as the accuracy in correctly classifying the 100 taps on the held out subject.

The linear SVM classification method produces a simple model of coefficient weights and an offset, which allows for easy transfer to the mobile application. Classification also can take place in near realtime, because of the computational simplicity.

### 2.7 Layperson Grading

Human subjects (*N* = 9, 2 Expert, 7 Novice) were recruited to validate the ability of laypeople to grade reflex responses accurately, and to compare to trained expert performance. Experts had both formal training and clinical experience in DTR assessment, for more information, see the Supplementary Material. Reflex grading took place in a virtual environment, with video training and evaluation. All training and testing took place in an online, remote format. Participants were first given a training video with three repeated examples of a tap and a response, the impact location of the tendon hammer on the bicep tendon was obscured in all cases. The rating scale employed was the National Institute of Neurological Disorders and Stroke (NINDS) 0–4 numerical scale. After reviewing the training video, participants were given 25 unlabelled tap videos to score, five for each of the five grades. Mean and median scores for each of the five were tabulated for each participant. All data collection took place under an institutionally approved protocol (GT# H20393).

## 3 Results

### 3.1 Expert and Novice Tendon Tapping Variability

Expert impact deceleration RSD was 18% while the novice acceleration RSD was 25%. The variability in acceleration indicates that similar levels of variability in tapping force should be expected for expert tendon tapping. Novice impact acceleration variability was larger than the expert’s, but still suitable for tendon reflex elicitation. Full results are shown in the Supplementary Material documentation.

### 3.2 SVM Classification Accuracy

The accuracy of the trained SVM model for the achilles tendon is shown in Table 1 Classification accuracy for each subject. Overall classification accuracy was 91.5% with a range of 67% when subject seven was held out for testing to 100% on subjects 1 and 6. With the relatively small subject pool, the high accuracy demonstrates the suitability of the SVM based classification method for determining tapping impact location from streamed (200 Hz) acceleration data.

**TABLE 1:** Classification accuracy for each subject. The bold is meant to emphasize the average location accuracy.

Subject	1	2	3	4	5	6	7	8	Mean
Location classification accuracy (%)	100	98	86	90	98	100	67	93	**91.5**

### 3.3 Layperson Grading Results

Aggregate results for each reflex grade from the reflex grading survey are shown in Table 2. The mean error across all seven of the novice participants and 25 reflex videos graded was 0.205 on the five point scale. However, mean error provides only one descriptor of the results. A more clinically relevant statistic may be the number of instances in which multiple trials would still result in an incorrect grade. In that case, taking the mean and median of all five trials for each participant results in an incorrect grade in just three and four of the 35 cases respectively. Both experts who completed the survey did not have any errors. Results are shown in Figure 2 and Table 2. There was a significant difference (p<0.05), between the number of errors in the 0 and 1 reflex grades and the 2 and 3 grades.

**TABLE 2:** Novice survey results by reflex grade.

Ground truth	0	1	2	3	4
Correct responses (%)	97	94	69	71	71
Median selection accuracy (%)	100	100	71	86	86
Mean selection accuracy (%)	100	100	86	86	86
Responses error ≤1 point (%)	100	100	100	100	97

## 4 Discussion

Taken together, the results shown demonstrate the potential viability of the remote deep tendon reflex exam. Tapping data on human subjects shows that 68% of the power difference between tapping locations is contained in acceleration signals below 100 Hz, and that the signals below 50 Hz are significantly different, allowing the combined use of readily available wireless IMUs and support vector machine classifiers to provide DTR elicitation feedback. The SVM classifier is shown to be capable of detecting tapping location on the Achilles tendon with 91.5% accuracy from streamed acceleration measurements enabling instant feedback to novices attempting to elicit reflex responses. With the developed application, remote users know when they have hit the tendon correctly, and can then grade the response.

Although novice performance is not perfect, the results indicate grading errors after a number of trials are relatively rare. Most importantly, out of a total of 175 novice graded reflexes, only a single response was more than 1 point away from the ground truth. The significant difference between errors in the 0 and 1 groups and the 2 and 3 groups indicates that areflexia or below normal reflexes are easier for novices to catch than the normal range. Although a larger sample size is needed, it is important to consider the range of conditions that can cause reflex responses to be on the lower end of the scale. Only a single reflex, the bicep tendon reflex, was evaluated, however it is expected that the novice performance in grading other reflexes would be similar.

Although this work has centered on the reflex exam being performed in a completely remote manner, with both tapping and grading done by novices, an important result emerged from the survey results. Both experts were capable of grading reflexes from video with 100% accuracy. An alternative procedure where the novice provides the elicitation via smart hammer and assistive application, and a video of the response is sent to the clinician may deserve further study and development. As the COVID-19 pandemic continues to dictate the use of telemedicine, this work provides experimental indications that remote implementation of the tendon reflex examination is possible.

## Data Availability

The raw data supporting the conclusions of this article will be made available by the authors, without undue reservation.
