# Supplementary material for: A Smart Tendon Hammer System for Remote Neurological Examination
Source: Front Robot AI. 2021 Mar 16;8:618656. doi: 10.3389/frobt.2021.618656 (PMC8008115; doi:10.3389/frobt.2021.618656)
Supplement: Supplementary file 1 [file datasheet1.pdf]

Supplementary Material

1 SMART HAMMER AND APPLICATION

The smart tendon hammer is shown in Figure S1. It contains a wireless inertial measurement unit (IMU) (MMC, mBient Labs, San Francisco, USA), and a standard Taylor reflex hammer (Niti-On, Japan). The application is shown in Figure S2. Both the application screen and the indicator light shown in Figure S1 turn green or red to indicate successful or incorrect tapping respectively. The icons and indicator light make it easy for novices to quickly recognize when they have performed the tapping incorrectly, regardless of where their attention is focused.

The hammer is manufactured by removing material from the silicone hammer head and inserting the plastic case containing the IMU. This material removal can be done by conventional milling, or through a stamping process with a thin metal tube of the appropriate diameter. Because of the elastic nature of the silicone, dimensional tolerances during machining are unimportant.

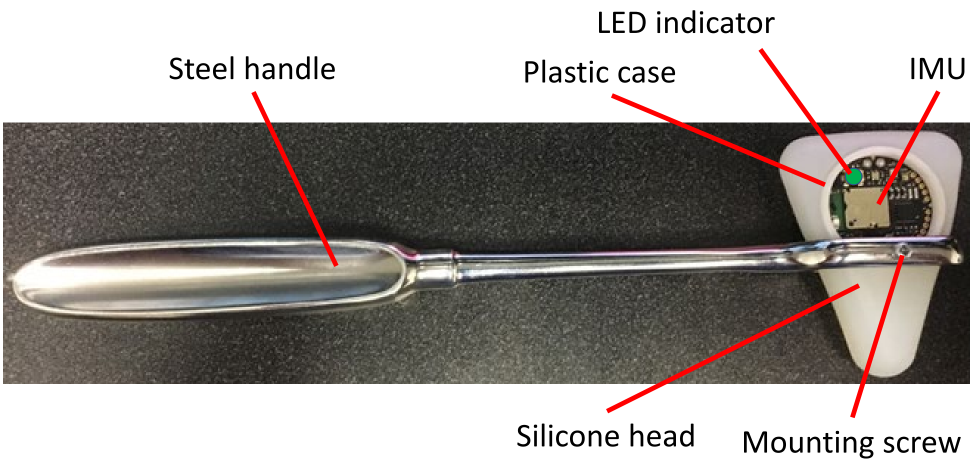

Figure S1. Picture of the smart tendon hammer used throughout this work.

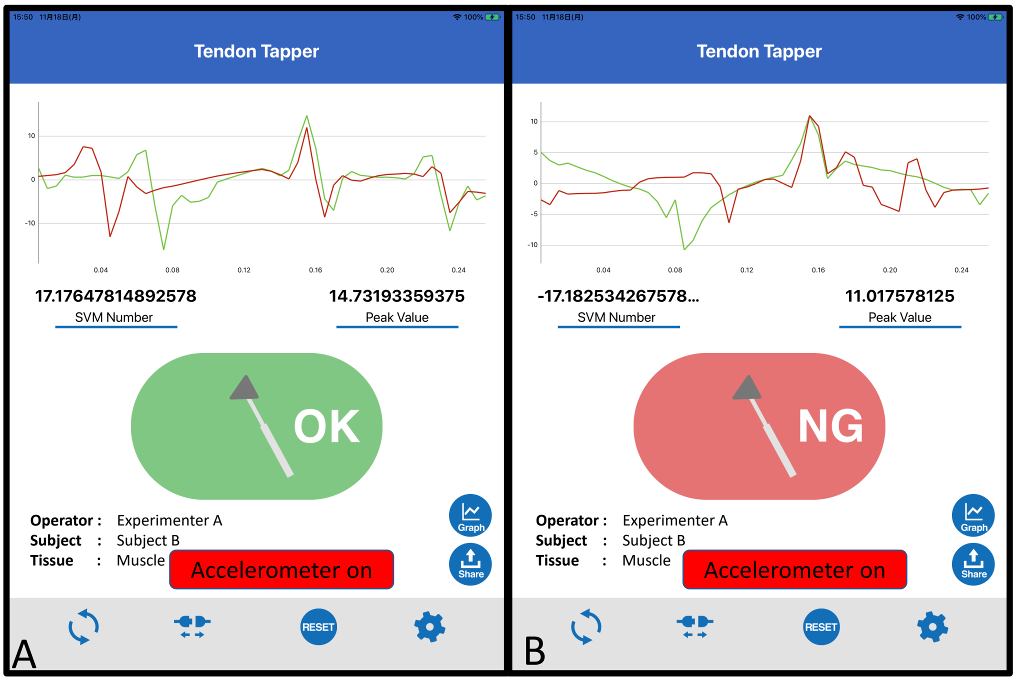

Figure S2. Tapping feedback and data collection application, A) Result after successful tap, B) Result after an incorrect tap, the red and green lines represent the acceleration of the most recent incorrect and correct taps respectively.

2 ACCELERATION MEASUREMENTS

Acceleration measurements in this work were made using the IMU shown in Figure S1. The sensor can be used to log data at up to 800Hz, or stream data directly from the sensor at up to 200Hz. Logged data must later be transmitted to the mobile device for offline processing, while streamed data can be processed online, as it is collected. Regardless of sample rate, data has a similar structure to the representative plots shown in Figure S3. The largest peak corresponds to the deceleration of the hammer during impact. The classification and frequency spectra results both come from these type of acceleration measurements. Tapping locations for the human Achilles experiments are shown in Figure S4. The same two locations were used in both the automated and manual tapping experiments.

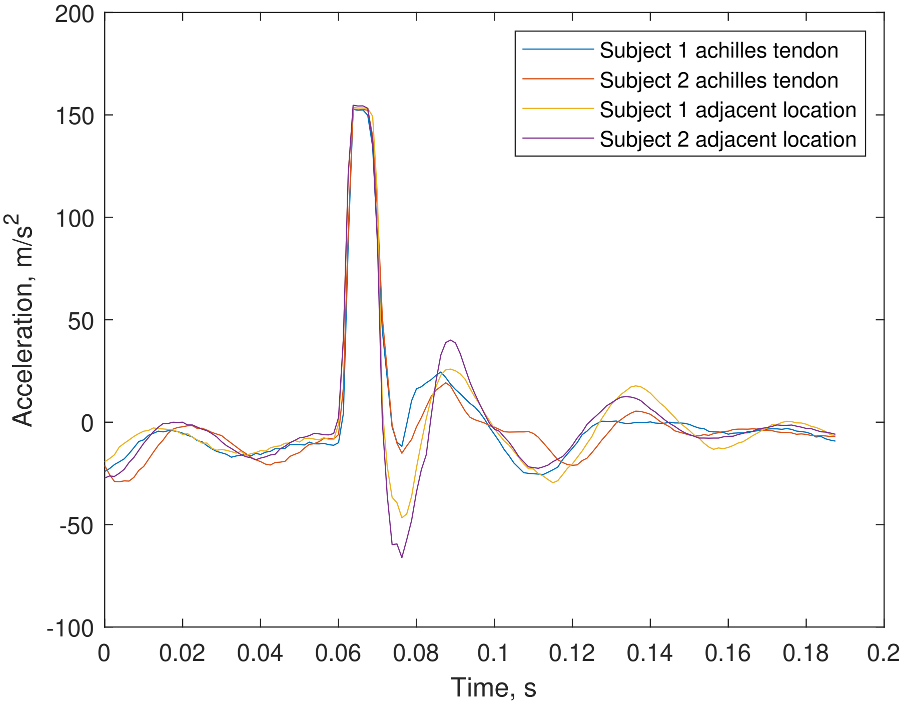

Figure S3. Representative acceleration time series for a tap to the Achilles tendon and adjacent location of both subjects.

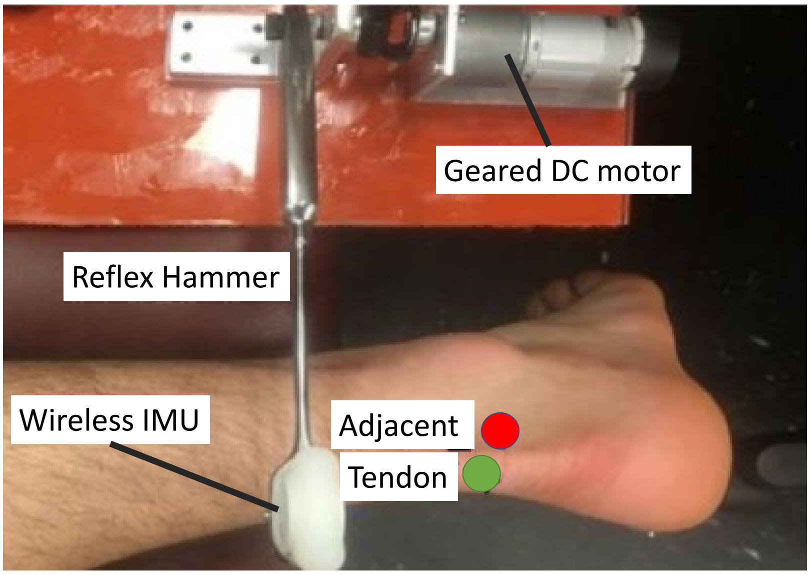

Figure S4. Experimental setup for automated tapping data collection on human subjects, the same locations were also used in the manual tapping experiments.

Results from the tapping experiments on human Achilles tendons are shown in Figure S5 and Figure S6. The frequency spectrum in Figure S5 provides a justification for the use of a 200 Hz accelerometer, as the majority of the signal power is in the lower frequency region. Additionally, Figure S6 shows the results of a χ^2 test comparing each 100 sample group for each frequency. Even with a conservatively corrected α, there are significant differences between the two locations at 4 frequencies, all below 100 Hz.

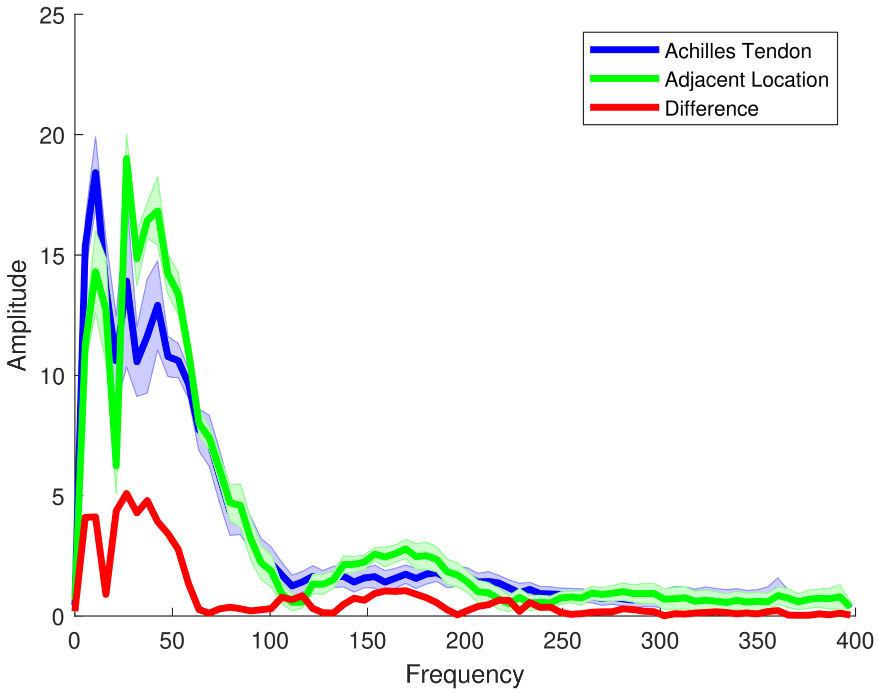

Figure S5. Mean amplitude spectrum of robotic tapping on two human subjects, shaded portions represent one standard deviation.

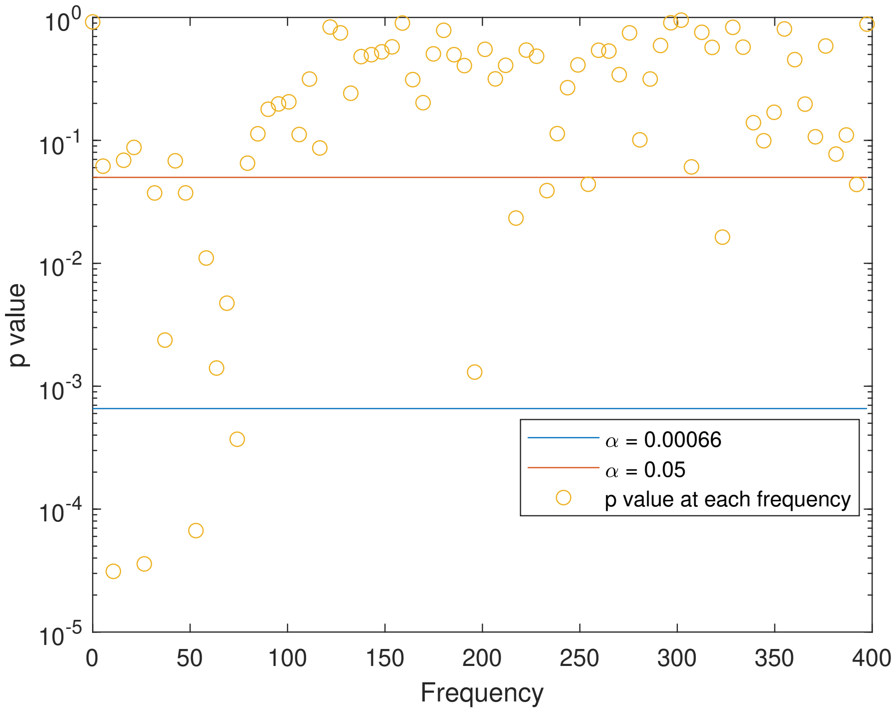

Figure S6. χ^2 associated p values for each frequency, computed via MATLAB command Chi2gof, the two values for α shown are the Bonferroni corrected value $(0.05/n)$ as well as the standard $\alpha = 0.05$.

3 TAPPING VARIABILITY

Tapping variability can be quantified in a number of ways. This paper is concerned only with measuring variability in the intensity of stimulation, as the reported classifier tracks location variability where it matters. In comparing tapping variability, the results in Figure S7 show comparable distributions between expert and novice accelerations, both before and during impact with the rubber tendon analog.

4 SVM BASED CLASSIFICATION

An example of the feature vector used for the SVM based classifier is shown in Figure S8. The feature vector consists of 51 acceleration points, 2 prior to peak deceleration, and 49 after the peak deceleration. Prior to training each of the features is standardized, other than this, the feature vectors consist of the raw acceleration data, without filtering or other feature extraction. Each tap collected from the human subjects corresponds with a feature vector used for training or testing. A total of 800 feature vectors were used, with 100 held out for testing each of the 8 classifiers.

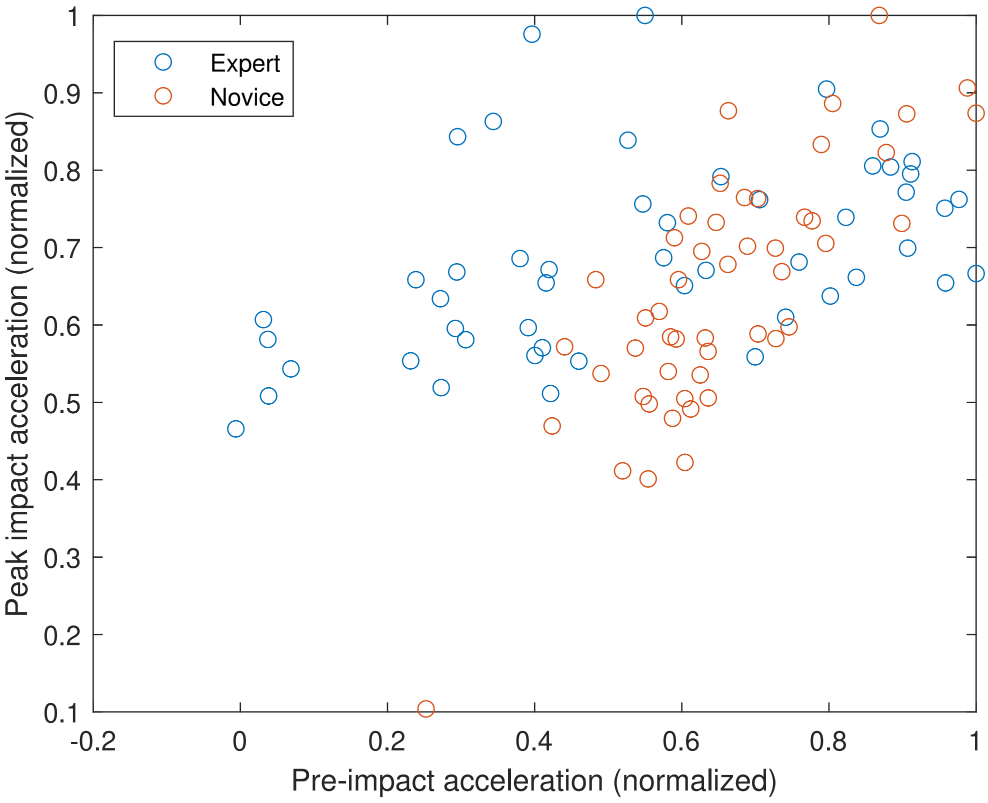

Figure S7. Expert and novice tendon tapping acceleration variability.

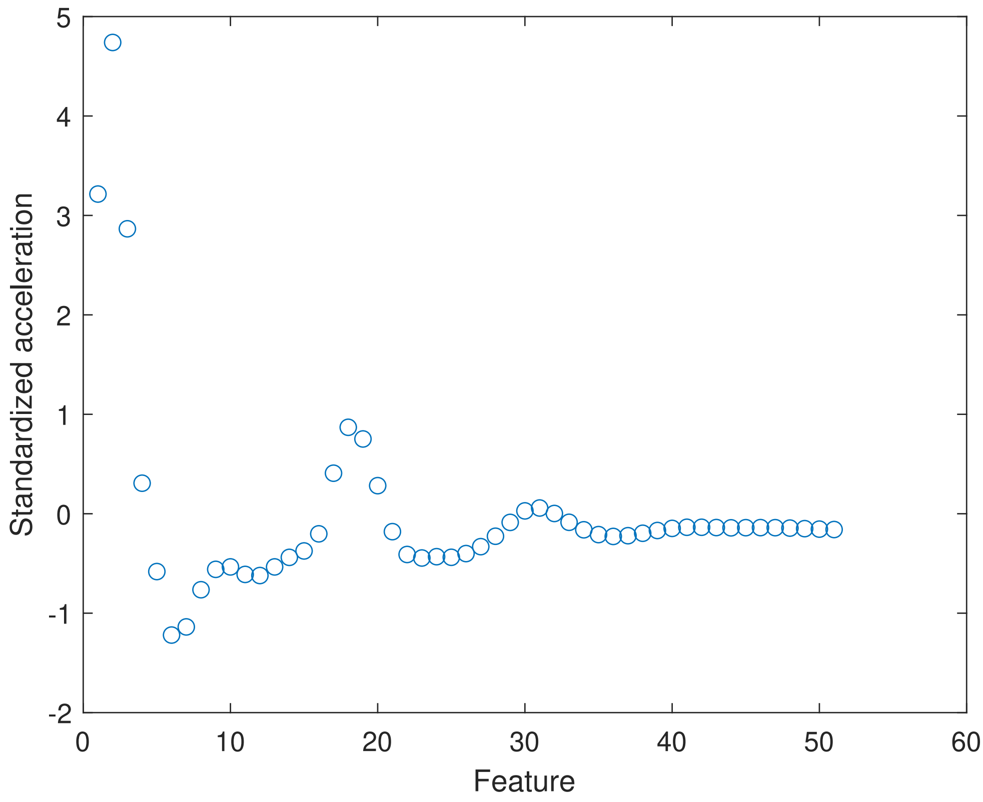

Figure S8. Representative feature vector used as training data for the SVM classifier.

5 REFLEX GRADING SURVEY

An example of the survey questions presented to research participants is shown in Figure S9. Both the training video with labeled responses and each of the unlabelled responses are shown in the supplementary video file. The survey consisted of a training video, in which subjects watched 3 labelled videos for each of the 5 scores, and a response section, in which they scored 25 unlabelled videos. Each survey question provided the 0-4 scale with descriptions from the NINDS scale.

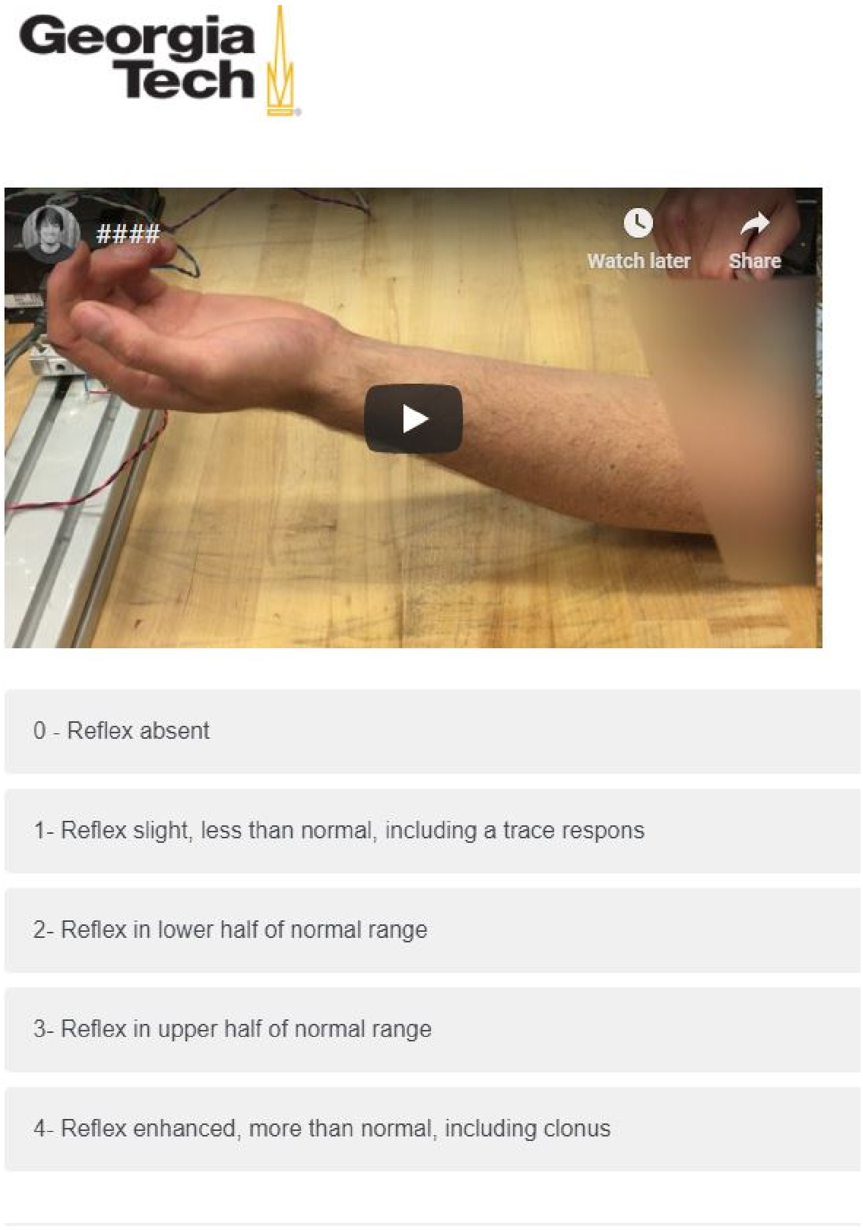

Figure S9. Example survey question with obscured hammer impact and 0-4 grading choices.
